# Highlight Removal of Multi-View Facial Images

**DOI:** 10.3390/s22176656

**Published:** 2022-09-02

**Authors:** Tong Su, Yu Zhou, Yao Yu, Sidan Du

**Affiliations:** School of Electronic Science and Engineering, Nanjing University, Nanjing 210046, China

**Keywords:** highlight removal, intrinsic image decomposition, multi-view images, facial image, specular reflection

## Abstract

Highlight removal is a fundamental and challenging task that has been an active field for decades. Although several methods have recently been improved for facial images, they are typically designed for a single image. This paper presents a lightweight optimization method for removing the specular highlight reflections of multi-view facial images. This is achieved by taking full advantage of the Lambertian consistency, which states that the diffuse component does not vary with the change in the viewing angle, while the specular component changes the behavior. We provide non-negative constraints on light and shading in all directions, rather than normal directions contained in the face, to obtain physically reliable properties. The removal of highlights is further facilitated through the estimation of illumination chromaticity, which is done by employing orthogonal subspace projection. An important practical feature of the proposed method does not require face reflectance priors. A dataset with ground truth for highlight removal of multi-view facial images is captured to quantitatively evaluate the performance of our method. We demonstrate the robustness and accuracy of our method through comparisons to existing methods for removing specular highlights and improvement in applications such as reconstruction.

## 1. Introduction

The removal of specular highlight reflection is an important problem in computer graphics, computer vision, and image processing since it provides useful information for the applications that need consistent object surface appearance [1], such as stereo reconstruction [2], visual recognition [3,4], augmented reality [5,6], object re-illumination [7] and dichromatic editing [8], many of which are multi-view issues. This is particularly significant in facial issues [9,10,11,12,13] since highlights are inevitable in facial images due to the oily skin surface of the human face and often show high intensity.

Nevertheless, previous highlight removal methods are typically based on a single image. The results of highlight removal can not be consistent between images from different viewpoints, even if some are improved for facial images [14,15,16]. In order to facilitate multi-view issues, the specular highlight reflections of multi-view images need to be removed consistently. Moreover, the extracted specular highlight can provide useful information for inferring scene properties such as surface normal and lighting directions.

To address this problem, we present a highlight removal method of multi-view facial images and jointly estimate the lighting environment. Based on the Lambertian consistency and mirror reflection model, respectively, we model the diffuse reflection and specular reflection of facial skin in three-dimensional space and render into each viewpoint by taking advantage of the prior about the geometry of the human face, which can be achieved via existing multi-view based 3D face reconstruction methods. Since the mirror reflection model is sensitive to the accuracy of the face geometry and the irregularities of the micro-facet structure, which is unable to characterize accurately limited by model precision, we further employ the dichromatic reflectance model and define the specular factor in each viewpoint. To obtain a physically reliable lighting environment, we provide non-negative constraints on light and shading in all directions, rather than the normal directions contained in the face. The removal of highlights is further facilitated by estimating illumination chromaticity, which is done by taking advantage of orthogonal subspace projection.

The main contributions of our study are summarized as follows:

- We propose a lightweight optimization solution for removing the specular highlight reflections of multi-view facial images and jointly estimate the lighting environment.
- We provide physically reliable intrinsic image properties through the non-negative constraints and the orthogonal subspace projection rather than priors.
- A dataset with ground truth for highlight removal of multi-view facial images is captured to quantitatively evaluate our method’s performance and demonstrate our method’s effectiveness.

**Figure 1:**
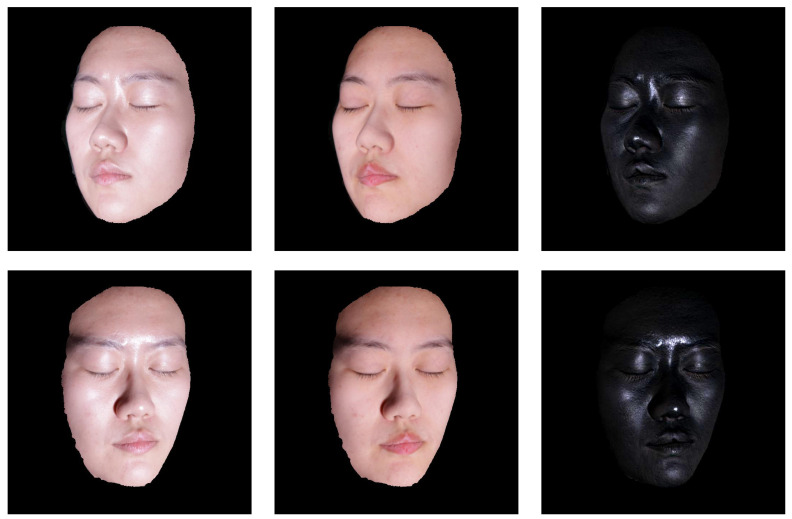
Examples of highlight removal of multi-view facial images. The first column presents facial images with highlights. The second and third columns show the corresponding highlight removal and specular images obtained through cross-polarization. Note that the images of the first two columns are not calibrated radiometrically for better visualization.

The rest of this paper is organized as follows: We commence with the motivation of our method and the related work in Section 2. In Section 3, we introduce the formulation and propose our method in Section 4. Section 5 contains the experiments of our method on laboratory captured images with ground truth highlight removal data, as shown in Figure 1. Finally, Section 6 concludes the paper.

## 2. Related Work

Highlight removal is a problem that has been studied for decades, as reviewed in [1,17]. Existing works for color images mostly originate from two categories: one is based on a single input image, and the other is based on multi-images. In this section, we briefly review previous works on highlight removal of general objects over these two categories and works particularly targeting facial images.

### 2.1. General Highlight Removal from a Single Image

Early approaches of general highlight removal from a single input image used color space analysis and treated an image pixel by pixel. Klinker et al. [18] analyzed the color histogram distributions using the convex polygon fitting technique and proposed methods linking color space with the dichromatic reflection model [19,20] to separate diffuse and specular reflections. Techniques that transformed images into other color spaces were later proposed [21,22,23,24]. The color space analysis was extended to spatial information analysis, enabling the handling of surface textures that can be inpainted [25] or that have a repetitive structure [26]. Tan et al. [27] introduced Maximum Chromaticity-Intensity Space to differentiate between the maximum intensity and maximum chromaticity. A pseudo-diffuse component image was created and later utilized to separate specular reflection from the image. Variants of this approach had employed a dark channel prior [28]. Yang et al. [29,30] treated the specular pixels as noise and used a bilateral filter for smoothing the maximum fraction of color components. An et al. [31] proposed the pure diffuse pixel distribution model. Many researchers have recently applied data-driven deep learning for single image highlight removal. Funke et al. [32] presented a GAN-based method for automatic specular highlight removal from a single endoscopic image. Lin et al. [33] proposed a fully-convolutional neural network that removed specular highlights from a single image by generating its diffuse component automatically and consistently. Wu et al. [34] presented Generative Adversarial Network by introducing the detection of specular reflection information as guidance.

A fundamental problem with methods based on a single image is that they either rely on image statistics or are based on strong prior assumptions. Therefore, such methods are not robust to changes in imaging objects, viewing angles, or lighting environments.

### 2.2. General Highlight Removal from Multiple Images

Multi-image methods use the information in an image sequence of the same scene, taken from different points of view or with different light information. Physically, the degree of light polarization can be considered a strong indicator of specular reflection, while diffuse is considered unpolarized. Therefore, many polarization-based methods with hardware assistance have been proposed, such as [35,36,37,38,39,40]. Specular highlights exhibit varying behaviors under different illumination directions or from different views [41]. Based on this property, multiple images based highlight removal techniques are proposed in the literature. Sato et al. [42] introduced temporal-color space analysis by using a moving light source. Lin et al. [43] used different illuminations for the same scene and then proposed linear basis functions for separating diffuse and specular components. Lin et al. [44] presented a method based on color analysis and multi-baseline stereo that used a sequence of images to achieve the separation of specular reflection. Prinet et al. [45] proposed the generation of specularity map from a video sequence. Wang et al. [46] used three cameras to take images of a transparent plastic package containing tablets.

Most closely related to our work is the method that utilizes multi-baseline stereo proposed by Lin et al. [44] to the best of our knowledge. Nevertheless, they assumed that scene points having specular reflection exhibit purely diffuse reflection in some other views, which is usually unsatisfactory in facial images.

### 2.3. Highlight Removal of Facial Images

Recently, several highlight removal techniques have been proposed, particularly targeting facial images. Li et al. [14] decomposed a single face photograph into its intrinsic components by utilizing human face priors, including statistics on skin reflectance and facial geometry. They also utilized a physically-based model called bidirectional surface-scattering reflectance distribution function to model skin translucency and the Planckian locus constraint for light color estimation. Later, Li et al. [15] improved the algorithm by adopting a skin model based on melanin and hemoglobin and an illumination model based on spherical harmonics. Unlike the optimization mentioned above, Yi et al. [10] proposed a deep neural network to extract faces’ highlights. The network is pre-trained with synthetic images and fine-tuned using an unsupervised strategy with real photos. Zhu et al. [16] adopted the structure of a conditional generative adversarial network to generate highlight-free images. Limited by the lack of diversity of training data, this method showed overall yellow tint results similar to the skin color of Asians.

The above methods, however, are also based on a single image and have the same limitations as general highlight removal methods based on a single image. That is, they are not robust to changes in viewing angles. As a result, they still cannot provide consistent intrinsic properties between images from different viewpoints to facilitate multi-view issues.

### 2.4. Our Work

**Figure 2:**
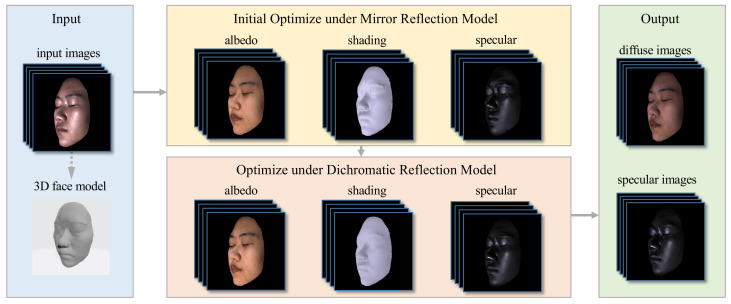
The schematic diagram of the proposed method. The left column shows the input multi-view images and the prior face geometry, which can be obtained roughly by input specular contaminated images as well. We first decompose the reflection components in 3D space under the Lambertian and mirror reflection models, respectively, and then render the result into 2D image space. The specular component is further pixel-wise optimized under the dichromatic reflection model in 2D image space. Parameters are estimated by minimizing the objective function using the Adam optimization algorithm [47]. The changes in the objective function determine the convergence criterion.

In this paper, we develop a lightweight optimization-based highlight removal method for multi-view facial images, as shown in Figure 2. Making use of the property that while the specular component varies with the viewing direction, the diffuse component reflects light equally in all directions, we can remove the highlight of multi-view facial images consistently between viewpoints and effectively reduce the ambiguity that exists in separating specular highlights from diffuse reflection occurs when the illumination chromaticity is similar to the diffuse chromaticity. Experiments show that our approach can provide superior performance and significantly facilitate multi-view issues such as 3D face reconstruction.

## 3. Formulation

According to the well-known dichromatic reflection model [48], the surface radiance is decomposed into a diffuse component and a specular one. Assuming that the image intensity *I* is calibrated to be linearly related to the image irradiance, which is also known as the surface radiance, the dichromatic reflection model can be expressed as follows:

(1) $$I_{j}=D_{j}+H_{j}.$$

Here, *j* indicates images of the *j*th viewpoint. *D* and *S* represent diffuse and specular reflection, respectively. We formulate the problem of highlight removal of multi-view facial images as decomposing a set of input facial images ${\left\{I_{j}\right\}}_{j=1}^{N}$ into their specular reflections ${\left\{H_{j}\right\}}_{j=1}^{N}$ and diffuse reflections ${\left\{D_{j}\right\}}_{j=1}^{N}$.

### 3.1. Illumination Model

Considering a distant lighting environment *L* with uniform illumination chromaticity *C* is incident on the face, we employ the spherical harmonics, which form a complete set of orthogonal functions on the surface of a sphere similar to the Fourier series on a circle, to model the angular distribution of lighting over the range of incident directions. Assuming that the diffuse reflection of the human face adheres to the Lambertian model, we can express *L* the coefficients $L_{lm}$ in spherical harmonic expansion as follow [49,50]:

(2) $$L\left(n\right)=\sum_{l,m}L_{lm}Y_{lm}\left(n\right).$$

Here, $Y_{lm}$ denotes the real form basis of the spherical harmonic function of degree *l* and order *m*, with l≥0 and −l≤m≤l, and *n* is a unit direction vector (surface normal, etc.).

### 3.2. Reflection Model

When a distant light reaches the surface of the human face at 3D position *p*, some portion of the light is reflected at the boundary, resulting in the specular reflection

(3) $$h\left(p,\omega_{o}\right)=\int_{\Omega }f_{s}\left(p,\omega_{i},\omega_{o}\right)CL\left(\omega_{i}\right)d\omega_{i},$$

and the rest is refracted into the facial skin and exits as diffuse reflection:

(4) $$d\left(p\right)=\int_{\Omega }f_{d}\left(p,\omega_{i},n_{p}\right)CL\left(\omega_{i}\right)\left(n_{p}\times \omega_{i}\right)d\omega_{i}.$$

Here, $f_{s}$ and $f_{d}$ are the bidirectional reflectance distribution functions (BRDF) which relate radiance exiting in direction $\omega_{o}$ to incoming light from direction $\omega_{i}$ for the specular reflection and diffuse reflection, respectively. $n_{p}$ is the surface normal vector at point *p*.

#### 3.2.1. Diffuse Reflection

As mentioned in Section 3.1, we assume that the diffuse reflection of facial skin adheres to the Lambertian model. As a result, the diffuse reflection at point *p* can be expressed as follows:

(5) $$d\left(p\right)=a\left(p\right)Cs\left(n_{p}\right).$$

Here, a(p) is the diffuse albedo of facial skin, which quantifies the fraction of incident light reflected by the skin surface, and $s(n_{p})$ is the geometric shading factor that governs the proportion of diffuse light reflected from the skin surface:

(6) $$s\left(n_{p}\right)=\int_{\Omega }L\left(\omega_{i}\right)\left(n_{p}\cdot \omega_{i}\right)d\omega_{i}.$$

According to [49], the diffuse shading $s(n_{p})$ can be represented by the coefficients $L_{lm}$ of the lighting environment in spherical harmonic expansion as well:

(7) $$s\left(n_{p}\right)=\sum_{l,m}{\hat{A}}_{l}L_{lm}Y_{lm}\left(n_{p}\right).$$

Here, the analytic formula for ${\hat{A}}_{l}$ has been derived in [51].

#### 3.2.2. Specular Reflection

To represent the specular reflection of facial skin, we first employ the physically based mirror reflection model as:

(8) $$h\left(p,\omega_{o}\right)=k\left(p\right)CL\left(\omega_{p}\right),$$

where *k* is the specular coefficient related to the Fresnel reflection coefficients, which in turn depend on the material refractive index [52]. $\omega_{p}$ is the incident direction of light which satisfied that the surface normal $n_{p}$ of pixel *p* is the half-angle direction of $\omega_{p}$ and the viewing direction $\omega_{o}$:

(9) $$\omega_{p}=2\langle n_{p},\omega_{o}\rangle n_{p}-\omega_{o}$$

The value of $\omega_{p}$ depends on changes in the viewpoint, and results in different specular reflections for different viewpoints, denoted as $h_{j}$ for *j*th viewpoint.

**Figure 3:**
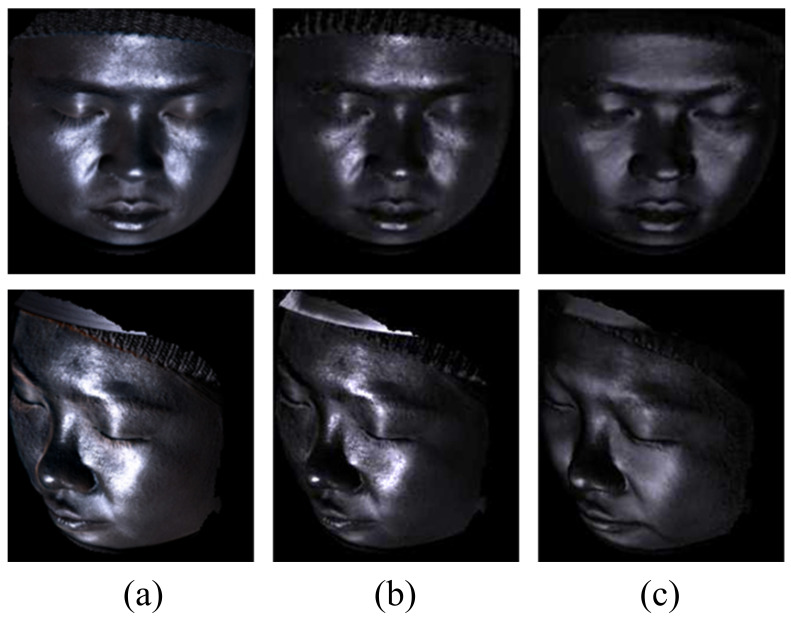
Examples of specular component. (**a**) Ground truth. Results by our method under (**b**) dichromatic model and (**c**) mirror reflection model.

Note that the mirror reflection model is sensitive to the accuracy of the face geometry and the irregularities of the micro-facet structure, which is unable to characterize limited by model precision. Moreover, the face geometry reconstructed from specular contaminated images is often inaccurate, especially in the region with high intensity around the nose and between the eyebrows, which is also the motivation of our method. In order to get rid of the dependency of the rough face geometry prior and remove the highlight specular more precisely, we take the result of the specular component under the mirror reflection model as initialization and further optimize the specular factor in the image space for each viewpoint under the dichromatic reflectance model. Figure 3 shows examples of specular reflection under two models.

According to the dichromatic reflection model, the specular component has the same chromaticity *C* as that of the light source [48]:

(10) $$H_{j}\left(x\right)=g_{j}\left(x\right)C$$

The pixel-wise parameter *g* is the corresponding of $k\left(p\right)L\left(\omega_{p}\right)$ and models the intensity of the specular reflection in each viewpoint. It is determined by not only the specular reflection coefficient but also the intensity of the light source that caused the specular reflection to the pixel *x*.

#### 3.2.3. Rendering

The reflection components can be rendered into each viewpoint,

(11) $$\begin{array}{c} H_{j}=R\left(m,n,c_{j},h_{j}\right)\\ D_{j}=R\left(m,n,c_{j},d\right) \end{array}$$

such as albedo, shading, and specular coefficient, if necessary. Here, R(·) is the traditional rasterizer rendering function that generates a rendered image from 3D properties, including triangle mesh *m*, mesh normals *n*, camera parameters *c* and per-vertex appearance (specular reflection, diffuse reflection, albedo, shading, etc). A sparse multi-view camera system is used to capture multi-view facial images, from which we obtain the above 3D properties by AgiSoft Metashape. Existing 3D face reconstruction algorithms from multi-view images such as [53,54,55] can also provide these parameters.

Substituting Equations (5), (8) and (11) into Equation (1), we can formulate the multi-view facial images as follows:

(12) $$I_{j}=D_{j}+H_{j}=R\left(m,n,c_{j},sa\right)+R\left(m,n,c_{j},h_{j}\right).$$

As a result, the highlight removal of multi-view facial images can be transformed into the reflection components separation of the human face in 3D space.

When we optimize the specular factor in the image space for each viewpoint under the dichromatic reflectance model, we can formulate the reflection model as:

(13) $$I_{j}=D_{j}+H_{j}=R\left(m,n,c_{j},sa\right)+g_{j}C,$$

by substituting Equation (5), (11) and (10) into Equation (1).

## 4. Multi-View Facial Images Highlight Removal

The objective function of our method for multi-view facial images highlight removal is:

(14) $$\mathrm{argmin}E_{0}+\lambda_{NN}E_{NN}+\lambda_{OSP}E_{OSP}$$

### 4.1. Data Term

The data term $E_{0}$ measures the difference between the reflectance model and the captured input images $I_{i}$:

(15) $$E_{0}^{1}\left(L_{lm},C,a,k\right)=\sum_{j=1}^{N}{∥R\left(m,n,c_{j},sa\right)+R\left(m,n,c_{j},h_{j}\right)-I_{j}∥}_{2}.$$

where the shading factor *s* is a function of $L_{lm}$ computed according to Equation (7).

When we separate the specular reflection for each viewpoint, the data term becomes:

(16) $$E_{0}^{2}\left(L_{lm},C,a,g\right)=\sum_{j=1}^{N}{∥R\left(m,n,c_{j},sa\right)+g_{i}C-I_{j}∥}_{2}.$$

### 4.2. Non-Negative Constraint Term

In this study, we employ several physically meaningful parameters, such as lighting environment, shading factor, specular coefficient, albedo, etc., which should bed strictly non-negative in all conditions.

As for parameters in the illumination model, while the shading tends to be positive in the normal direction of the human face, it could be negative in other directions, as in the lighting environment. Thus, we compute the lighting environment and shading factor in all directions:

(17) $$\begin{array}{c} L_{0}=\sum_{l,m}L_{lm}Y_{lm}\left(n_{0}\right)\\ s_{0}=\sum_{l,m}{\hat{A}}_{l}L_{lm}Y_{lm}\left(n_{0}\right) \end{array}$$

Here, $n_{0}$ is the mesh normals of a unit sphere.

Together with the albedo and specular coefficient, we define the non-negative constraint (NN) term as follows:

(18) $$E_{NN}^{1}\left(L_{lm},C,a,k\right)={∥L_{0}∥}_{2}+{∥s_{0}∥}_{2}+{∥a∥}_{2}+{∥k∥}_{2}$$

Similarly, when we separate the specular reflection for each viewpoint, the non-negative constraint term becomes into:

(19) $$E_{NN}^{2}\left(L_{lm},C,a,g\right)={∥L_{0}∥}_{2}+{∥s_{0}∥}_{2}+{∥a∥}_{2}+{∥g∥}_{2}$$

### 4.3. Orthogonal Subspace Projection Term

By adopting the orthogonal subspace projection (OSP) [56], the radiance of a specular contaminated image can be projected onto two orthogonal subspaces. One is parallel, while the other is orthogonal to the light chromaticity *C*. Based on the theory of matrix projections, we can design the orthogonal projector:

(20) $$P=E-CC^{T}/∥C∥$$

where *E* is the identity matrix. Substituting Equation (8) or Equation (10) into Equation (1), and then multiply both sides of equation by projector *P*, yields the same equation:

(21) $$PI_{j}=PD_{j}+PH_{j}=PD_{j}$$

As a result, the specular component is removed, while the diffuse component is preserved at the cost of losing one dimension of information:

(22) $$E_{OSP}\left(L_{lm},C,a\right)={∥PI_{j}-PD_{j}∥}_{2}={∥PI_{j}-PR\left(m,n,c_{j},sa\times C\right)∥}_{2}.$$

This term measures the difference between the reflectance model and the captured input images $I_{j}$ under orthogonal subspace projection, which leads to constraining the illumination chromaticity.

### 4.4. Optimization

We use PyTorch to implement the model and minimize the objective function of Equation (14) sequentially by using the Adam optimizer [47]. The initial learning rate is 1 × 10^−2^, and the number of training times is 20 epochs. The optimization is iterated until the change in the objective energy falls below the threshold 1 × 10^−3^. We set the value of the regularization weights $\left\{\lambda_{NN},\lambda_{OSP}\right\}$ to $\left\{10,1\right\}$ in our experiments.

We initialize the illumination as white ambient light, where *C* equals $\left[1/\pi ,1/\pi ,1/\pi \right]$ and the spherical harmonics coefficients are zero for all values except for $L_{00}=1$, that is ambient lighting only. The albedo *a* is initialized by the color of the 3D face model divided by *C*, and the specular coefficients *k* are all zero.

We first estimate the parameters $\left\{L_{lm},C,a,k\right\}$ based on the mirror reflection model by minimizing:

(23) $$\underset{L_{lm},C,a,k}{\mathrm{argmin}}E_{0}^{1}+\lambda_{NN}.E_{NN}^{1}+\lambda_{OSP}E_{OSP}$$

After that, we render the result of *k* into each viewpoint by Equation (11) and take the rendered images ${\left\{K_{j}\right\}}_{j=1}^{N}$ as the initialization of the pixel-wise parameter *g*. The specular component is further optimized by minimizing:

(24) $$\underset{L_{lm},C,a,g}{\mathrm{argmin}}E_{0}^{2}+\lambda_{NN}.E_{NN}^{2}+\lambda_{OSP}E_{OSP}$$

## 5. Results

In this section, experiments are performed to evaluate the proposed method. Firstly, we introduce the Laboratory Captured Dataset and the FaceScape dataset [57] used in the experiments. Then, we test our method and show quantitative results and visual effects compared with the previous methods. After that, we demonstrate the ablation studies of our method. Lastly, we evaluate our method’s improvement in several applications.

### 5.1. Dataset

#### 5.1.1. Laboratory Captured Dataset with Ground Truth

The input multi-view facial images are captured under a sparse multi-view camera system containing a set of metal brackets, 12 DSLR cameras (Canon EOS 80D), a photography lighting. Four-column brackets in arc shape are used to mount cameras. In the center of the arc, we place a forehead bracket accessory (equipment of computer refractometer) to fix the position of the human face to capture the stable and high-precision facial skin appearance.

We use a classic 24-color chart (X-Rite ColorChecker) to radiometric calibrate the camera system linearly related to the image radiance. The polarizers are placed in front of the cameras and the light source. A non-metal dielectric spherical ball is used to correct the polarizers. So that we set the cross and parallel polarization and filter out the highlight when capturing pictures so that pairs of real facial photos with and without highlights can be collected. As a result, the ground truth highlight removal results are obtained through cross-polarization for each viewpoint.

AgiSoft Metashape software developed by AgiSoft LLC is used to generate initial 3D properties, including triangle mesh *m*, mesh normal *n*, and camera parameters *c*. Existing multi-view-based 3D face reconstruction algorithms [53,54,55] can also obtain these 3D properties.

We recruited 20 Asian participants (17 male, 3 female) and captured 20×12 linear raw images. The effective resolution is 6024×4020. By taking advantage of 3D properties, we resize and crop the original image data with a window of 800×400, preserving the information of the face. For data augmentation, we apply the image flip. All participants had fully read and understood the informed consent form and signed a portrait rights authorization agreement. All data are only used for non-profit academic research areas.

#### 5.1.2. FaceScape Dataset

To qualitatively analyze the performance of our approach, we employ the FaceScape Dataset [57], an open-source 3D face dataset consisting of multi-view facial images, camera parameters and 3D face models with a texture map. We resize and crop the image data using the same laboratory-captured dataset strategy.

### 5.2. Experiments

#### 5.2.1. Comparisons

Quantitative comparisons of multi-view highlight removal on the Laboratory Captured Dataset are presented in Table 1. We compare the specular highlight removal results of our method using either the mirror reflection model (M.) or dichromatic reflection model (D.) to three existing techniques [29,58,59]. In order to show comparisons for both absolute intensity errors and structural similarities, we use the root-mean-square error (RMSE) (smaller is better, denoted as ↓), and the structural similarity (SSIM) [60] (larger is better, denoted as ↑) for error measurement. Note that the RMSE is rescaled by 100 for better viewing. The best results are shown in bold. According to Table 1, our method obtains highlight removal images closer to the ground truth than other methods. Our method under the mirror reflection model can obtain good results when each viewpoint shares the same parameters, while the method under the dichromatic model is pixel-wise optimized in each viewpoint to obtain more accurate results.

In order to explore the consistency of results from different viewpoints, we calculate the standard deviation (SD) and relative standard deviation (RSD) of our method and three existing techniques [29,58,59], as shown in Table 2. Note that the SD RMSE is also rescaled by 100 for better viewing. Regarding the robustness in changes of viewpoints, our methods show superior improvement to methods from a single image. It is worth noting that, in the mirror reflection model, we use the same parameters to ensure the consistency of the results under different viewpoints, which is also well maintained after changing into the dichromatic reflection model.

**Figure 4:**
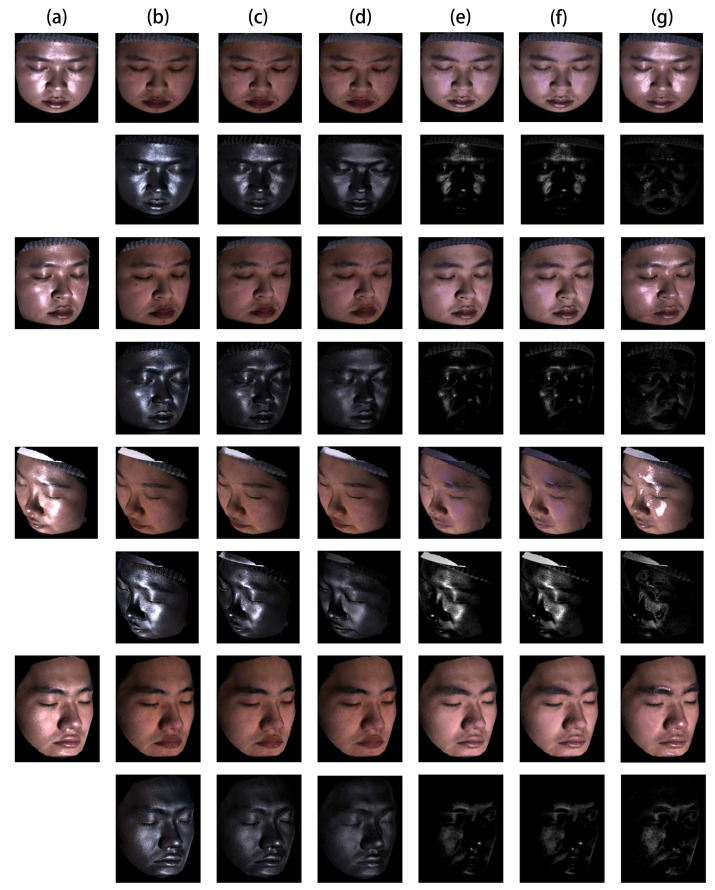
Qualitative evaluation of highlight removal on the Laboratory Captured Dataset. (**a**) Input images. (**b**) Ground truth diffuse and specular components obtained by cross-polarization. (**c**–**g**) Separated diffuse and specular components by (**c**) our method based on dichromatic reflection model, (**d**) our method based on mirror reflection model, (**e**) [58], (**f**) [59], and (**g**) [29]. The specular component is rescaled by two for better visualization.

**Figure 5:**
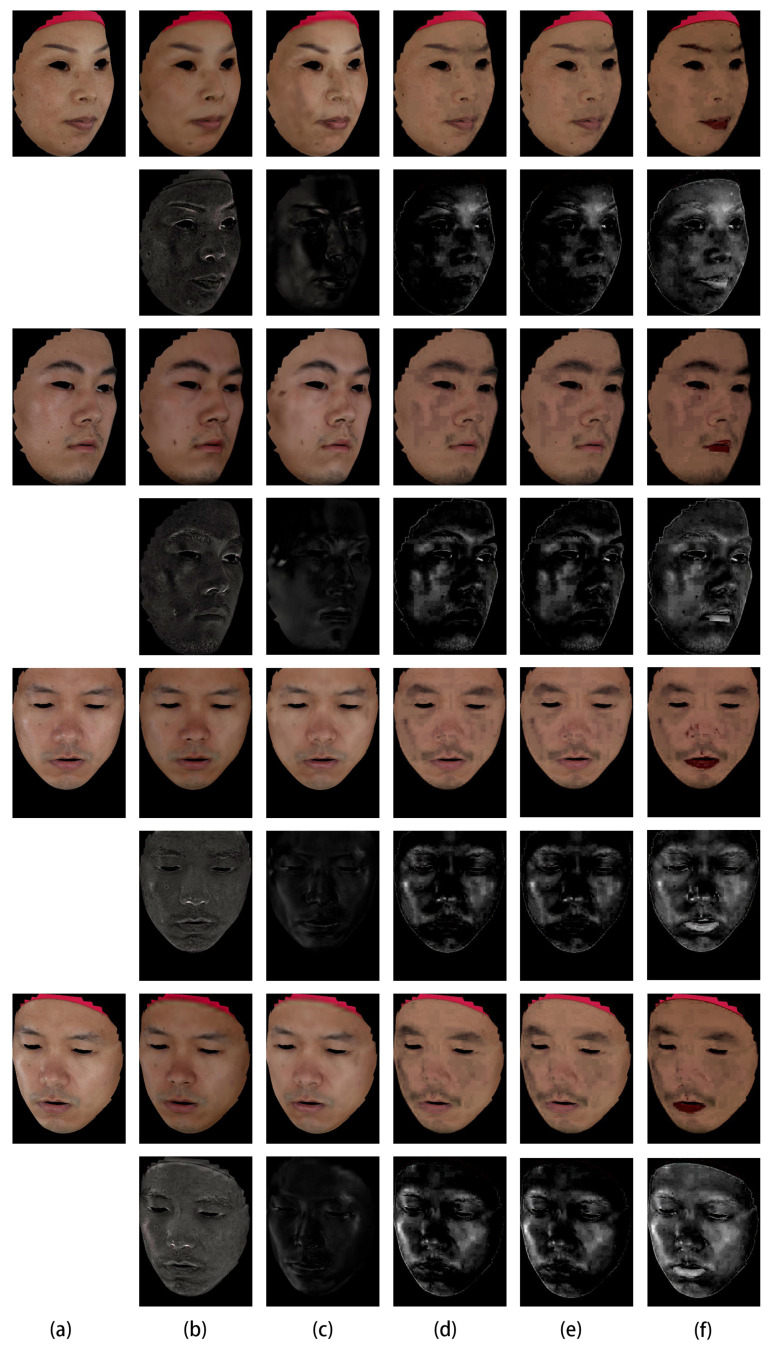
Qualitative evaluation of highlight removal on FaceScape Dataset. (**a**) Input images. (**b**–**f**) Separated diffuse and specular components by (**b**) our method based on dichromatic reflection model, (**c**) our method based on mirror reflection model, (**d**) [58], (**e**) [59], and (**f**) [29]. The specular component is rescaled by two for better visualization.

As for images in Figure 4 and Figure 5, we qualitatively analyze the performance of our method on the laboratory captured and FaceScape dataset. As shown in the first four rows of Figure 4 and the last four rows of Figure 5, the results of [29,58,59] are not consistent with different viewing angles since they are relying on a single input image. The specular reflections still exist in the results, especially in the region around the cheek and forehead. Benefiting from the multi-view information, we can process facial images with large-area, high-intensity specular highlights and obtain results robust to changes in viewing angles. As shown in Figure 4c,d and Figure 5b,c, our results based on mirror reflection show significant improvement and exhibit further improvement based on the dichromatic reflection model. By making use of the advanced pixel-wise optimization, our method based on the dichromatic reflection model can separate the specular highlights as much as possible while preserving the view-independent diffuse reflection initialized by the mirror reflection model and offset the effects caused by the inaccuracy of the face geometry and the irregularities of the microfacet structure.

#### 5.2.2. Ablation Studies

Our method has two optimization steps, and the objective function of each step consists of data term, non-negative constraint term, and orthogonal subspace term. These terms have similar but different formulas and share the same weights for different optimization stages. In the initial optimization step, view-independent parameters help the optimization obtain consistent diffuse reflection. The pixel-wise optimization step helps offset the effects caused by the face geometry’s inaccuracy and the microfacet structure’s irregularities. The orthogonal subspace projection term is used to constrain the chromaticity of light, while the non-negative constraint term is used to guide the optimization to maintain stability in physical properties. The weights of these terms are adjusted according to our experiments.

**Figure 6:**
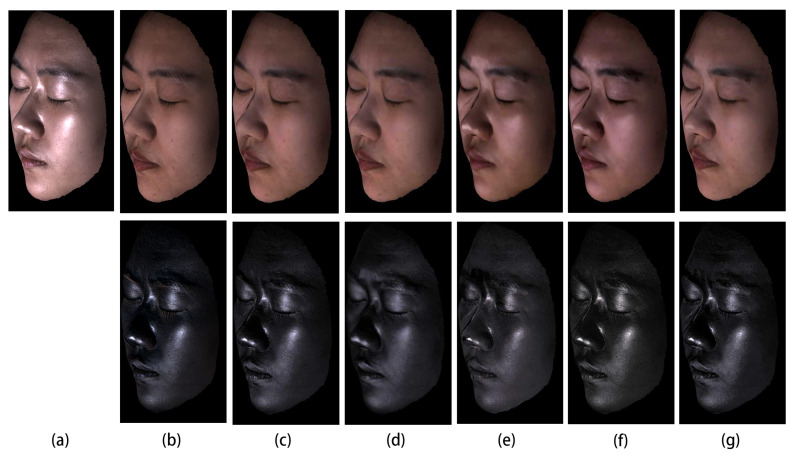
Ablation studies of the proposed multi-view highlight removal on the Laboratory Captured Dataset. (**a**) Input image. (**b**) Ground truth. (**c**) Ours with full loss. (**d**) Ours only with the initialization by the mirror reflection model. (**e**) Ours without initialization by the mirror reflection model. (**c**–**e**) are setted with $\lambda_{NN}=10$ and $\lambda_{OSP}=1$. (**f**) Ours with $\lambda_{OSP}=0.1$. (**g**) Ours with $\lambda_{NN}=1$. The specular component is rescaled by two for better visualization.

Table 3 and Figure 6 demonstrate the results of the ablation studies of our work. It shows that the complete loss function leads to better results. As for the regularization weights, minor weight for the orthogonal subspace projection term makes the light chromaticity worse. If the weight for the non-negative constraint term is too small, many areas will be filled with dark colors.

**Figure 7:**
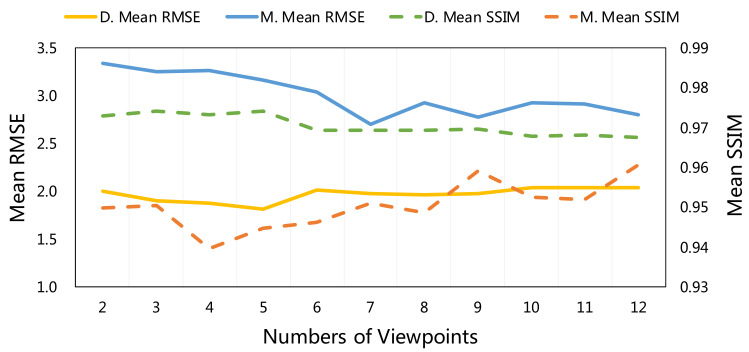
Comparison of experimental results on the number of viewpoints.

The experiment on the number of images is necessary since we use multi-view images. We experiment on one set of faces whose error is above the median since the combinations of the number of viewpoints is too large. Table 4 and Figure 7 demonstrate the mean RMSE (R.) and SSIM (S.) of our methods under a different number of viewpoints. With the increase in the number of viewpoints, our method under the mirror reflection model improves slightly, while the method under the dichromatic reflection model basically remains the same. It is worth mentioning that our method shows better performance than other methods when only two viewpoints are used.

#### 5.2.3. Applications

**Figure 8:**
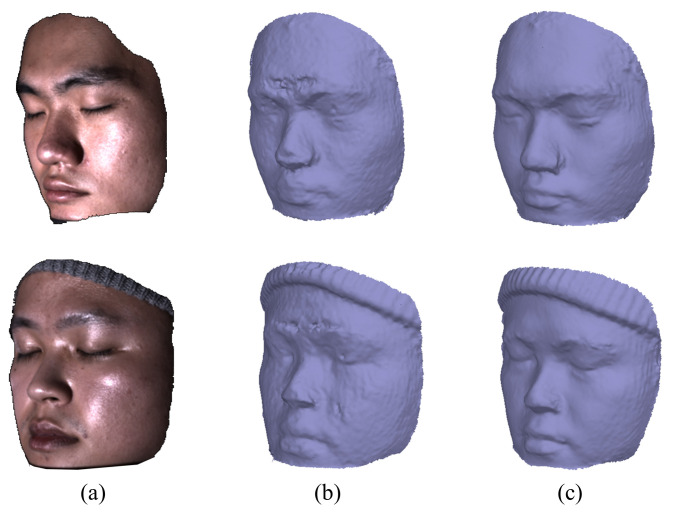
Improvement in the 3D face reconstruction. (**a**) A sample of input images. (**b**) Face reconstruction results obtained by highlight-contaminated facial images. (**c**) Face reconstruction results obtained by the highlight removal results of our method.

Since the motivation of our method is to facilitate multi-view issues, we demonstrate the face reconstruction performance using the result of our method as shown in Figure 8. The reconstructed face geometry using images highly affected by specular highlight is shown in Figure 8b, which is also the rough prior of face geometry used in our method. The face model reconstructed using the highlight removal images by our method in Figure 8c shows significant improvement, especially in the region with high intensity around the nose and between the eyebrows. Note that the improvement of reconstruction can also facilitate our highlight removal method of multi-view images.

**Figure 9:**
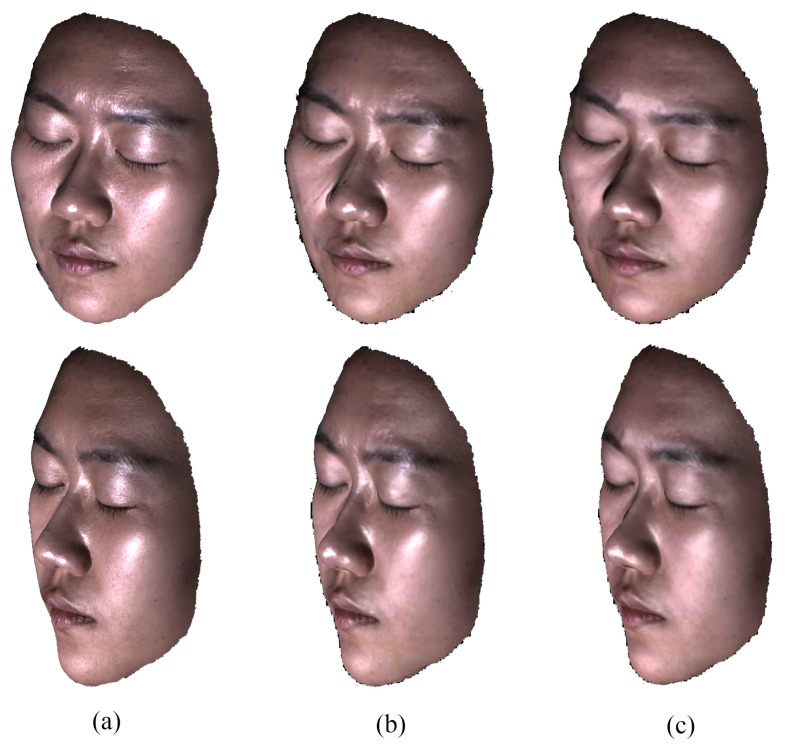
Experiment of synthesizing novel views. (**a**) Ground truth. (**b**) Synthesizing results using two input viewpoints. (**c**) Synthesizing results using eleven input viewpoints.

Moreover, we demonstrate the potential of our method under the mirror reflection model for synthesizing novel views using a sparse set of input viewpoints. The experimental results with different numbers of input viewpoints are shown in Figure 9, which produce photorealistic rendering at new viewpoints.

## 6. Conclusions

This paper presents a lightweight, optimized-based highlight removal method for multi-view facial images. The proposed method can facilitate many applications, such as 3D face reconstruction from multi-view images, re-lighting, and AR effects on the face image. One particular problem for multi-view facial issues such as 3D face reconstruction we addressed here is that the presence of highlights makes the color of the same point inconsistent between images from different viewpoints. We proposed a lightweight, optimized-based framework for multi-view facial images to solve this problem. We took the view-independent diffuse results achieved under the mirror reflection model as initialization. We performed pixel-wise optimization in each viewpoint using non-negative constraints and orthogonal subspace projection.

In our experiments, we showed that our method could separate the specular highlights as much as possible while preserving the view-independent diffuse reflection and offset the effects caused by the inaccuracy of the face geometry and the irregularities of the microfacet structure. We captured a dataset with ground truth for the highlight removal of multi-view facial images to evaluate quantitative performance. A qualitative evaluation of the publicly available FaceScape dataset was also performed. In the last, we showed robustness in the number of input images and improvements for the reconstruction task.

Future research could be combined with specific facial problems for joint optimization, such as reconstructing the face model with the results of highlight removal and, in turn, improving the highlight removal results. In addition, collecting more facial image datasets, especially with ground truth, is essential to obtain better results.

## Figures and Tables

**Table 1:** Quantitative evaluation of multi-view highlight removal on the Laboratory Captured Dataset.

Method	RMSE		SSIM	
	Mean ↓	Median ↓	Mean ↑	Median ↑
[29]	4.4932	4.4276	0.9287	0.9323
[59]	5.9070	5.5094	0.8953	0.9294
[58]	5.8736	5.4703	0.8961	0.9304
Ours (M.)	2.0048	1.6245	0.9788	0.9808
Ours (D.)	**1.7220**	**1.5618**	**0.9800**	**0.9823**

**Table 2:** Standard deviation and relative standard deviation of multi-view highlight removal.

Method	RMSE		SSIM	
	SD ↓	RSD ↓	SD ↓	RSD ↓
[29]	0.0668	1.4864	1.6285	1.7534
[59]	0.3230	5.4681	8.7117	9.7304
[58]	0.3239	5.5141	8.7467	9.7611
Ours (M.)	0.0404	2.0133	0.6412	0.6551
Ours (D.)	**0.0202**	**1.1722**	**0.5962**	**0.6063**

**Table 3:** Ablation Studies of multi-view highlight removal on the Laboratory Captured Dataset.

	Mean RMSE	Median RMSE	Mean SSIM	Median SSIM
Full loss	**1.7220**	**1.5618**	**0.9800**	**0.9823**
Only M. initialization	2.0048	1.6245	0.9788	0.9808
Without M. initialization	2.2306	2.1598	0.9652	0.9671
Without OSP term	1.8530	1.8179	0.9801	0.9805
Without NN term	1.8986	1.8587	0.9781	0.9785

**Table 4:** Quantitative evaluation of the experiment on the number of viewpoints on the Laboratory Captured Dataset.

Number of Viewpoints	Ours (D.)		Ours (M.)	
	RMSE	SSIM	RMSE	SSIM
2	2.0061	0.9730	3.3379	0.9499
3	1.9013	0.9740	3.2522	0.9505
4	1.8797	0.9733	3.2629	0.9397
5	1.8168	0.9740	3.1570	0.9446
6	2.0133	0.9693	3.0350	0.9462
7	1.9738	0.9694	2.6980	0.9511
8	1.9635	0.9695	2.9307	0.9486
9	1.9703	0.9696	2.7753	0.9590
10	2.0406	0.9678	2.9241	0.9527
11	2.0354	0.9681	2.9136	0.9519
12	2.0409	0.9675	2.7964	0.9606

## Data Availability

Not applicable.
